# Effects of a chronotype-adapted diet on weight loss, cardiometabolic health, and gut microbiota: study protocol for a randomized controlled trial

**DOI:** 10.1186/s13063-024-07996-z

**Published:** 2024-02-28

**Authors:** Monica Dinu, Sofia Lotti, Giuditta Pagliai, Antonia Napoletano, Marta Tristan Asensi, Ilaria Giangrandi, Rossella Marcucci, Amedeo Amedei, Barbara Colombini, Francesco Sofi

**Affiliations:** 1 Department of Experimental and Clinical Medicine, School of Human Health Sciences, University of Florence, Largo Brambilla 3, Florence, 50134 Italy; 2 Unit of Clinical Nutrition, Careggi University Hospital, Florence, Italy; 3 Atherotrombotic Diseases Unit, Careggi University Hospital, Florence, Italy

**Keywords:** Chronotype, Diet, Weight loss, Cardiometabolic health, Gut microbiota, Randomized controlled trial

## Abstract

**Background:**

Obesity and its associated health complications have become a global public health concern, necessitating innovative approaches to weight management. One emerging area of research focuses on the influence of chronotype, an individual’s preferred timing for daily activities, on eating habits, weight regulation, and metabolic health. Recent observational studies suggest that the misalignment between an individual’s chronotype and external cues, such as meal timing, may contribute to metabolic dysregulation and obesity, but evidence from intervention studies is still limited. This study protocol describes a randomized controlled trial designed to explore the effects of a chronotype-adapted diet, compared with a diet with a conventional calorie distribution, on weight loss, cardiometabolic health, and gut microbiota composition.

**Methods:**

A total of 150 overweight/obese adults will be recruited for this 4-month parallel-group, randomized, two-arm, open-label, superiority trial with 1:1 allocation ratio. Participants will be randomly assigned to either the intervention group or the control group. The intervention group will receive a low-calorie chronotype-adapted diet with a calorie distribution adapted to the individual chronotype (morning or evening), optimizing meal timing according to their peak metabolic periods. The control group will follow a standardized low-calorie healthy eating plan without considering chronotype. Both diets will have equivalent daily calorie content, adjusted according to gender and starting weight. Anthropometric measurements, body composition, blood, and fecal samples will be obtained from each participant at the beginning and the end of the study. The primary outcome is weight change from baseline. Secondary outcomes are changes from baseline in body mass index (BMI), fat mass, lipid and glycemic profile, fecal microbiota profile, and short-chain fatty acids (SCFAs).

**Discussion:**

The results of this randomized controlled trial have the potential to advance our understanding of the complex interactions between chronotype, diet, body weight, and health outcomes. By providing evidence for personalized dietary interventions based on individuals’ circadian preferences, this research could offer insights into personalized nutrition strategies. Such knowledge could guide the development of innovative dietary interventions to optimize the prevention and management of overweight and obesity, while also improving the risk profile of these individuals.

**Trial registration:**

ClinicalTrials.gov NCT05941871. Registered on 18 May 2023.

**Supplementary Information:**

The online version contains supplementary material available at 10.1186/s13063-024-07996-z.

## Introduction

In recent years, the prevalence of overweight and obesity has reached epidemic proportions [1], posing significant challenges to public health worldwide [2]. The complex interplay between lifestyle factors, including diet, physical activity, and sleep patterns, has been recognized as key contributors to weight management and overall health [3]. However, effective strategies for the prevention and management of overweight and obesity are still limited [3].

An emerging body of evidence suggests that the timing of food intake, in relation to individual chronotype, may be involved in modulating metabolic processes and promoting optimal body weight and cardiometabolic health [4–6]. Chronotype refers to an individual’s preference for activity and sleep patterns during a 24-h cycle [7] and is generally determined based on the scores obtained from validated questionnaires such as the Morningness Eveningness Questionnaire (MEQ) [8]. Early risers, who are preferentially active in the morning, are said to have a morning chronotype, while late risers with more nocturnal activities are said to have an evening chronotype [7]. Research has indicated that chronotype is not merely a preference but is genetically determined and associated with variations in circadian rhythms, hormonal regulation, and metabolic function [9, 10].

The rationale for examining the effects of a chronotype-adapted diet stems from the growing number of studies suggesting that circadian rhythms play a crucial role in metabolic regulation and overall health [11–13]. Disruptions in circadian rhythms, often resulting from modern lifestyle factors such as irregular sleep schedules and food intake, have been associated with adverse health outcomes, including obesity, metabolic syndrome, and alterations in gut microbiota composition [14, 15]. A chronotype-adapted approach involves consuming most calories during the peak period of wakefulness, when metabolic processes are most active, and adapting the timing of meals accordingly for individuals with different chronotypes. A recent clinical study conducted in overweight and obese subjects showed that a chronotype-adapted dietary intervention resulted in greater weight and fat mass loss than a non-adapted intervention, suggesting that aligning mealtimes and macronutrient distribution to an individual’s chronotype may be a promising approach to enhance weight loss efforts [16]. In contrast, several studies have shown that individuals with a misaligned sleep–wake schedule, especially late risers forced into an early-rising routine, experience disruptions in various physiological processes, including alterations in appetite regulation, glucose metabolism, and lipid profiles [12, 17]. These metabolic disorders can ultimately lead to weight gain and an increased risk of cardiometabolic diseases. The composition and activity of the gut microbiota have also been linked to energy metabolism and metabolic health [18]. In this regard, recent investigations have demonstrated bidirectional communication between the gut microbiota and the host’s circadian system, further emphasizing the potential role of chronotype, dietary patterns, and timing in modulating this intricate relationship [15].

Despite the great interest in this topic, most of the studies available to date are observational. Therefore, we propose to conduct a randomized controlled trial to investigate the effects of a chronotype-adapted diet, compared with a diet with a conventional calorie distribution, on weight loss, cardiometabolic health, and gut microbiota composition. Both dietary interventions will be standardized to ensure equivalent daily calorie content, adjusting for gender, and starting weight.

We hypothesize that aligning dietary patterns with the individual inherent biological clock will optimize metabolic processes and promote beneficial changes in gut microbial composition, ultimately leading to improved health outcomes. We also speculate that a chronotype-adapted diet may have a lower dropout rate because these diets are more easily adapted to each patient’s preferences.

## Methods: participants, interventions, and outcomes

### Study design and setting

**Fig. 1:**
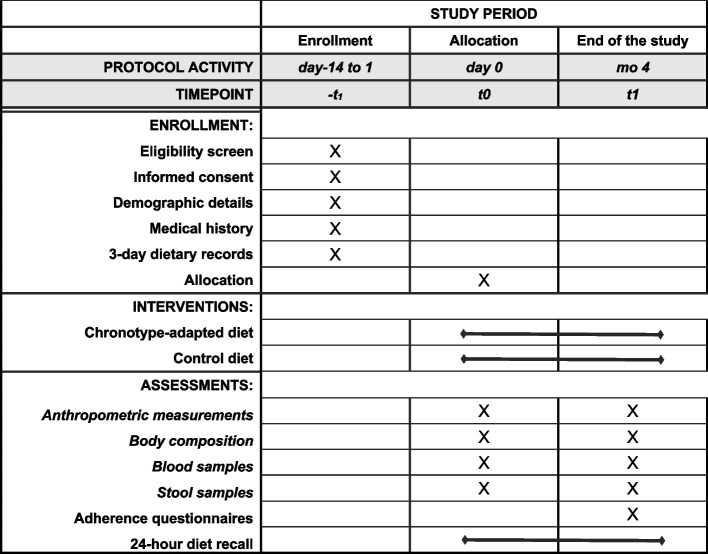
SPIRIT figure reporting the phases of the trial and data collection time points

A 4-month parallel-group, randomized, two-arm, open-label, superiority trial with 1:1 allocation ratio to either intervention or control arms will be conducted at the Unit of Clinical Nutrition of the Careggi University Hospital, Florence, Italy. The study design follows the SPIRIT reporting guidelines [19] (see Fig. 1 and Supplementary file 1).

In the design of the study protocol, there was no direct involvement from the public or patients. While we recognize the growing importance of patient and public involvement in research, for this study, the protocol was developed within the framework of scientific and clinical expertise.

### Participants

A total of 150 overweight and obese females and males aged between 18 and 65 years with a body mass index (BMI) ≥ 25 kg/m^2^ will be included in the study. Exclusion criteria will be the presence of chronic illnesses or unstable conditions that could interfere with the dietary intervention (e.g., cancer, kidney or liver disease, inflammatory bowel disease, cognitive decline, and psychiatric disease); drug therapies that could interfere with the effectiveness of the dietary intervention (e.g., use of corticosteroids, and anti-diabetic drugs); pregnancy or intention to become pregnant in the next 12 months; breastfeeding; current or recent (last 3 months) adoption of a low-calorie diet.

Before starting any research activities, including the initial run-in period, the study investigators will obtain written informed consent from each participant. All subjects must voluntarily agree to participate and will be free to withdraw from the study at any time. Study participants will also be asked for written informed consent to keep the biological samples for future research and to publish the data anonymously.

### Interventions

After a 2-week run-in period, eligible participants will be divided into two groups: the intervention group, which will be given a low-calorie chronotype-adapted diet with a calorie distribution adapted to the individual chronotype (morning or evening), and the control group, which will be given a low-calorie control diet with a standard calorie distribution.

In the intervention group, chronotypes will be assessed according to scores obtained with the Morningness-Eveningness Questionnaire (MEQ) [8]. Participants with a score between 52 and 86 will be classified as morning chronotypes and will receive a morning-adjusted diet, while those with a score between 16 and 51 will be classified as evening chronotypes and will receive an evening-adjusted diet [16]. The daily caloric distribution will be as follows: (1) morning chronotype − 50% of the daily kcal will be administered before lunch and 15% in the second part of the day (specifically: 40% at breakfast, 10% in the morning snack, 35% at lunch, 5% at snack and 10% at dinner); (2) evening chronotype − 15% of the daily kcal will be administered before lunch and 50% in the second part of the day (specifically: 10% kcal at breakfast, 5% in the morning snack, 35% at lunch, 10% at snack and 40% at dinner). The daily caloric distribution in the control group will be as follows: 20% kcal at breakfast, 10% in the morning snack, 35% at lunch, 10% at snack, and 25% at dinner.

Both diets will be hypocaloric with respect to the participants’ energy requirements. Specifically, they will provide 1200 kcal for women and 1400 kcal for men with a body weight < 90 kg at the first visit, and 1400 kcal for women and 1700 kcal for men with a body weight > 90 kg. Furthermore, both dietary profiles will have about 30% of the energy coming from fat, 15–20% from protein, and the remaining 50–55% from carbohydrates (mainly complex). The only difference will be the distribution of calories throughout the day, which will be chronotype-based in the intervention group and standard in the control group. Participants may discontinue the intervention or withdraw from the study for the following reasons: (1) at the request of the participant; (2) if the investigator considers that a participant’s health will be compromised due to adverse events or concomitant illness that develop after entering the study.

To improve adherence to intervention protocols, qualified nutritionists will provide participants with a detailed 1-week menu plan based on their intervention group, with all food ingredients expressed by weight and/or volume, and a handout containing details of the assigned diet with possible substitutions. No meals or supplements will be provided. Participants will prepare their own meals or eat at a restaurant. For both diets, alcoholic beverages will be limited to one per day for women and two per day for men. Participants will be asked not to change their exercise habits during the study. In addition, the use of cortisone therapies, antidiabetic drugs or the use of vitamin supplements, minerals, or products aimed at weight loss will not be allowed during the study. Patients who need to start using these products under a doctor’s recommendation during the intervention period will be excluded from the study. No further arrangements or ancillary assistance will be provided upon completion of the study.

### Outcomes

The primary outcome is weight change from baseline. Secondary outcomes are (1) body mass index (BMI) and fat mass change from baseline; (2) lipid and glycemic profile change from baseline (total cholesterol, LDL-cholesterol, HDL-cholesterol, triglycerides, fasting glucose); and (3) fecal microbiota profile and short-chain fatty acids (SCFAs) change from baseline. Body weight will be measured as a unique and established quantitative primary outcome, while assessment of body composition, biochemical profile, and gut microbiota will provide information on the cardiometabolic status of participants. The metric used will be the change in mean values from the beginning to the end of each dietary intervention.

### Participant timeline

**Fig. 2:**
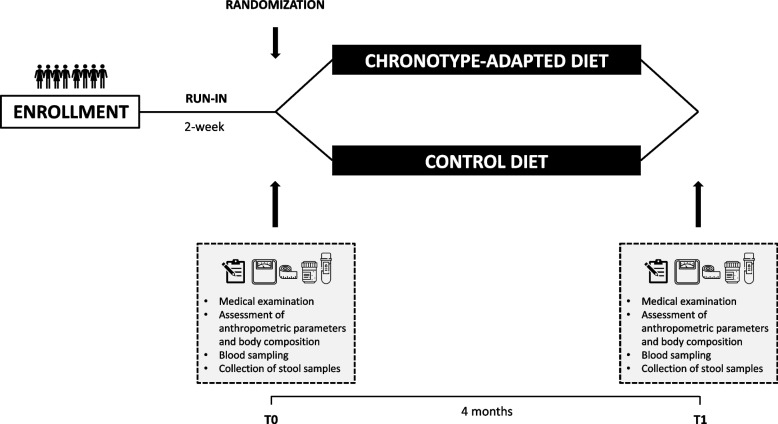
Organization of the intervention study

Study timeline is depicted in Fig. 2. Before starting the intervention, a 2-week run-in period will be implemented during which participants will be asked to complete a 3-day dietary record (two weekdays and a weekend day). A qualified nutritionist will analyze all 3-day dietary records using the Metadieta software application (Me.Te.Da., San Benedetto del Tronto, Italy), which is linked to the Italian database of food composition (INRAN/IEO, 2008). Then, participants will be randomly divided into two groups, each assigned to the intervention or control group. There will be two clinical evaluations of the study population: at the beginning (*Time 0*) and at the end of dietary interventions (*Time 1*).

### Sample size

The calculation of the sample size and statistical power was measured considering the 80% probability to show as statistically significant (*p* < 0.05) a 1.4% weight reduction difference between the intervention group and the control group, with a standard deviation of 0.4% and a loss at follow-up of 22%. This calculation was derived from a study in the literature that evaluated the difference between a diet adapted to the chronotype and a control diet in overweight/obese subjects [16]. The minimum number of subjects to be recruited to obtain reliable results with a statistical power of 0.8 (beta) and 0.05 (alpha) is 54 participants per group. Dropouts will be included in the intention-to-treat analysis, which includes all participants initially randomized. Per-protocol analyses, focusing on those who complete the trial without major deviations, will also be conducted to provide a comprehensive perspective on treatment efficacy.

### Recruitment

Female and male participants will be recruited using advertisements on local media, newspapers, social media, official papers, and websites. We will also recruit from our existing database of participants and friends or relatives of the hospital and university staff.

### Assignment of interventions: allocation

A web-based online randomization procedure will be used to generate the allocation sequence. No adaptive randomization procedures will be performed. The random assignment sequence will be produced and managed by an investigator who will not participate in the recruitment of participants. The order of assignment will be kept secret from the experimenters who will enroll participants or assign interventions. The group assignment will be presented on a folded sheet of paper in a sealed envelope.

### Assignment of interventions: blinding

Blinding of study participants and nutritionists will not be possible because of the obvious differences between intervention diets; however, outcome measures cannot be easily influenced by the observer. In addition, study personnel who will enroll participants, data collectors, outcome assessors, and data analysts will be blinded to the treatment assignment, and a researcher from outside the research team will enter the data into the database.

### Data collection and management

Assessments and data collection at baseline and follow-up will be carried out at the Unit of Clinical Nutrition of the Careggi University Hospital, Florence, Italy, by study staff. All subjects will be examined between 7:30 am and 10:30 am after a 12-h fasting period. The standardized assessment at baseline for the intervention and control groups will include a questionnaire regarding demographic information, risk factors, and comorbidities. In addition, during the baseline visit, participants will be educated about the objectives and methods of the clinical study. Sessions for all participants will be similar in duration and content, except for details about diet; nutritionists will be instructed not to comment in favor of one diet over the other or indicate their eating habits.

Height will be measured with a stadiometer, while weight will be assessed with a professional weighing scale with 0.1-kg accuracy (TANITA, model TBF-410). BMI will be calculated as weight (kg)/height (m)^2^. Body composition will be determined with a bioelectrical impedance analyzer (Akern, model SE 101) at baseline and at follow-up visits. Blood samples will be collected at the beginning and end of each intervention phase. Blood samples will be centrifuged at 3000 rpm for 15 min, aliquoted to obtain serum, and stored at – 20 °C until analysis. The following biochemical measures will be evaluated: complete blood count, lipid profile (total cholesterol, LDL cholesterol, HDL cholesterol, triglycerides), fasting glucose, liver function tests (aspartate aminotransferase (AST), alanine transaminase (ALT), gamma-glutamyl transferase (γGT), renal function tests (serum creatinine, urea, uric acid), mineral profile (sodium, potassium, magnesium, calcium), iron metabolism (iron, ferritin), and vitamin profile (vitamin B12, folic acid).

Stool samples (four or five scoops totaling 4 g) will be collected before and after each intervention. Stool sample collection kits, including containers, will be provided to each participant. Fecal microbiota profiles and SCFAs (acetic, butyric, isobutyric, propionic, valeric, and isovaleric acids) will be evaluated. Total microbial DNA will be extracted from the faces by repeated scouring. The V3 and V4 hypervariable regions of the 16S rRNA gene for bacteria and ITS1-4 for fungi will be sequenced on the Illumina MiSeq platform, following the Illumina protocol for preparing 16S metagenomic sequencing libraries. SCFAs will be extracted using aqueous NaOH containing an internal standard. After extraction, an aliquot of supernatant fecal water will be derivatized with a propanol/pyridine mixture. The organic extract will be analyzed by gas chromatography-mass spectrometry (GC–MS) using deuterated internal standards and an appropriate GC Wax column.

Plans to promote participant retention and follow-up completion include the use of behavior change strategies such as self-monitoring and availability of study staff for any dietary counseling. In addition, participants will be contacted once during the study period to promote dietary compliance. A nutritionist will call unannounced, and each participant will recall his or her 24-h diet. At the end of the study, participants will be asked to fill out an adherence questionnaire in which they will indicate the extent to which they complied with the diets they received, their mealtimes and daily calorie distribution. They will also have to indicate whether and what kind of modifications they made to the diets they received and whether they encountered any difficulties.

If participants do not show up for the scheduled appointment, a maximum of three phone calls will be made and an e-mail will be sent before withdrawing the participant from the study. Participants who wish to prematurely discontinue the study before the 4-month evaluation will not undergo further clinical and laboratory evaluations. The reason for withdrawal will be documented in the participant’s record for later analysis when interpreting the results. The entire study will be discontinued if the observed results warrant premature discontinuation.

All data will be collected in an electronic database. Data or other identifiable documents will not be recorded in the database, and participants will be identified only by a unique study identification number. Hard copies of the files linking the participant identification number to the person’s contact details will be stored securely in a locked file cabinet in a locked office, accessible only to key members of the research team. Participant files and other source data (including copies of protocols, questionnaires, original test result reports, correspondence, informed consent records, and other documents related to the conduct of the study) will be retained for the maximum period allowed by the institution. The data will be made available upon request after publication.

Several strategies will be used to improve data quality during collection, including careful recruitment, a structured and time-limited protocol, inclusion of a run-in period, limiting the burden and inconvenience of data collection for participants, developing a trusting and collaborative relationship between research units and participants, and double data entry. Storage of biological samples will be under appropriate conditions according to standard methods. Blood samples will be aliquoted and stored at – 20 °C for 4 years before being used or destroyed. Preserved samples will be used only for research purposes with the consent of the donor. Destruction of samples will be properly documented.

### Statistical analysis

Statistical analyses will be performed using SPSS software for Macintosh (SPSS Inc., Chicago, IL, USA). Data will be analyzed using intention-to-treat and on-treat procedures. The primary outcome (change in body weight) will be analyzed within each group using Student’s *t*-test for paired data. The difference in absolute change (mean value at baseline subtracted from the mean value after the intervention for each subject) will be estimated using the Student’s *t*-test for independent data. Evaluation of distributions and checking for outliers will be performed using histograms and box plots. If appropriate, variables will be logarithmized to normalize the distributions of data. Continuous variables that follow a normal distribution will be summarized using the mean and standard deviations. Categorical variables will be presented in terms of frequencies and percentages. After testing the regression assumption, a general linear model for repeated measurements with adjustments for possible confounding factors will be run to compare the effects of different interventions. Data for the general linear model will be reported as geometric means and 95% confidence intervals. The same analyses will be performed for secondary outcomes. Subgroup analyses will be performed to analyze possible differences in the changes, according to some characteristics of the study population, such as age and gender.

Before starting the data analysis, the level and possible causes of missing data will be investigate using appropriate tables. This information will be used to determine whether the level and type of missing data have the potential to introduce bias into analysis results or substantially reduce the precision of the estimates for the proposed statistical methods. Sensitivity analyses will be performed, based on the assumption that the missing outcomes are the worst or best possible in the different randomization groups. If these show that the conclusions may differ based on the missing values, further multiple imputation will be performed for the missing values. These analyses will consider the results of any losses at follow-up to the extent that they relate to differences in the measured variables (i.e., under the assumption of random missingness). A *P* value < 0.05 will be considered statistically significant.

### Oversight and monitoring

Regular monitoring of this study will be conducted by both the research team and the local Institutional Review Board. Given the study’s limited objectives, short duration, and the low-risk nature of the intervention, the establishment of a Data Monitoring Committee and a Trial Steering Committee was deemed unnecessary. The research group, appointed by the principal investigator, comprises a study coordinator, two physicians, four nutritionists, and a biostatistician. The team will convene regularly, typically monthly, to comprehensively assess ongoing trial progress. This includes a thorough review of study advancements, recruitment rates (actual versus predicted), data quality and return rates, protocol amendments, and general research study matters. The principal investigator will oversee these meetings, developing the agenda and serving as the study’s primary contact. At the halfway point and conclusion of the study, the protocol team will furnish the local Institutional Review Board with a comprehensive monitoring report, encompassing a review of activities, progress, challenges, and any pertinent issues of concern.

Any changes to the protocol and information provided to participants will be submitted to the Ethics Committee for approval prior to implementation. No interim analysis will be conducted. Access to data will be limited to qualified personnel with unique password-protected accounts. Adverse events, such as unfavorable and undesirable signs, symptoms, abnormal laboratory results, or illnesses temporally associated with the intervention diet, will be collected from the time of randomization until the final 4-month follow-up visit for each participant, whether or not they are considered related to the intervention study. All adverse events will be followed up until their resolution.

## Discussion

Human biological processes are rhythmically regulated, as seen in the sleep/wake or hunger/satiety rhythms [20]. Although previous observational studies have postulated a link between the endogenous circadian rhythms, dietary habits, and the risk of developing obesity and other chronic diseases [21], the complex interaction between time of ingestion, chronotype, body weight, and specific gut microbiota communities involved in the modulation of the circadian rhythm is still unclear. Given the growing burden of obesity worldwide, gaining new insights into the possible mechanisms that condition the variable response to food intake and weight loss diets is critical to improving the effectiveness of weight management programs.

The results of this trial have the potential to significantly contribute to the existing body of knowledge on chrono-nutrition. If the chronotype-adapted diet proves effective in promoting weight loss and improving cardiometabolic health outcomes, it may pave the way for personalized dietary approaches that consider the individual inherent sleep–wake pattern. Such tailored interventions could be an important component of precision medicine and hold promise for more effective weight management strategies. In addition, investigating the relationship between chronotype, diet, and gut microbiota composition could offer valuable insights into the mechanisms underlying the observed effects. The gut microbiota plays a crucial role in host metabolism and energy balance, and alterations in its composition have been associated with metabolic disorders [18]. Understanding how chronotype influences gut microbial populations and their metabolic activity may provide novel therapeutic targets for promoting metabolic health and preventing obesity-related diseases.

One of the strengths of this study protocol is the randomized controlled trial design, which allows for rigorous assessment of causal relationships. Randomization helps minimize confounding factors and improves internal validity, enabling us to draw more reliable conclusions about the effects of chronotype-adapted diet. In addition, the inclusion of comprehensive outcome measures, including weight loss, body composition, metabolic markers, and gut microbiota composition, provides a holistic understanding of the potential impacts of the dietary intervention. Despite its potential contributions, this study has several limitations that should be acknowledged. Firstly, the study duration and sample size may limit the generalizability of the findings. Secondly, although the chronotype-adapted diet may demonstrate greater efficacy, the extent remains uncertain. Our study was designed with the statistical power to demonstrate a minimum difference of at least 1.4% between intervention and control groups, a value determined by standard practices for sample size estimation in power analysis. However, it is noteworthy that this chosen value may not necessarily have clinical relevance. Additionally, our ability to predict the sustainability of weight loss beyond the study duration is limited due to the exploratory nature of the research. Given the typical decline in diet adherence after the initial 6 months, uncertainties persist regarding long-term effects. Finally, the reliance on self-reported data, such as chronotype assessment and dietary intake, introduces the possibility of recall bias. If this trial leads to highly relevant results, future studies could incorporate objective measures such as actigraphy and dietary biomarkers, which are more accurate but have much higher costs.

In conclusion, the results obtained from the implementation of this trial have the potential to advance our understanding of the complex interactions between chronotype, diet, and health outcomes. By providing evidence of personalized dietary interventions based on an individual’s circadian preferences, this research may contribute to the development of innovative dietary approaches to optimize the management of overweight and obesity, while also improving the risk profile of these patients.

## Trial status

The study has received all necessary regulatory approvals. The currently approved version of the protocol is 2.0 (version date 08/11/2022). Recruitment began on March 1, 2023, and the completion date is scheduled for April 30, 2024.

### Supplementary Information

**Supplementary Material 1.**

## Data Availability

Only the research team will have access to the final trial dataset. The principal investigator must ensure that participants’ privacy is respected. Data and source documents will be kept in such a way that they can be accessed later for monitoring or inspection purposes by the Ethics Committee. At the end of the study, participants will be able to request a copy of the study results from the principal investigator. The final report will follow the CONSORT 2010 guidelines. Study results will be submitted for publication in a peer-reviewed journal. Plans for dissemination to national and international conferences will be discussed with the researchers prior to implementation.

## Abbreviations

ALT: Alanine transaminase
AST: Aspartate aminotransferase
BMI: Body mass index
DNA: Deoxyribonucleic acid
γGT: Gamma-glutamyl transferase
HDL: High-density lipoprotein
LDL: Low-density lipoprotein
RNA: Ribonucleic acid
SCFAs: Short-chain fatty acids
